# Malonyl-CoA Accumulation as a Compensatory Cytoprotective Mechanism in Cardiac Cells in Response to 7-Ketocholesterol-Induced Growth Retardation

**DOI:** 10.3390/ijms24054418

**Published:** 2023-02-23

**Authors:** Mei-Ling Cheng, Cheng-Hung Yang, Pei-Ting Wu, Yi-Chin Li, Hao-Wei Sun, Gigin Lin, Hung-Yao Ho

**Affiliations:** 1 Department of Biomedical Sciences, College of Medicine, Chang Gung University, Taoyuan City 33302, Taiwan; 2 Healthy Aging Research Center, Chang Gung University, Taoyuan City 33302, Taiwan; 3 Metabolomics Core Laboratory, Healthy Aging Research Center, Chang Gung University, Taoyuan City 33302, Taiwan; 4 Clinical Metabolomics Core Laboratory, Chang Gung Memorial Hospital at Linkou, Taoyuan City 33302, Taiwan; 5 Graduate Institute of Biomedical Sciences, College of Medicine, Chang Gung University, Taoyuan City 33302, Taiwan; 6 Department of Medical Imaging and Intervention, Chang Gung Memorial Hospital at Linkou, Taoyuan City 33302, Taiwan; 7 Imaging Core Laboratory, Institute for Radiological Research, Chang Gung University, Taoyuan City 33302, Taiwan; 8 Department of Medical Imaging and Radiological Sciences, Chang Gung University, Taoyuan City 33302, Taiwan; 9 Department of Medical Biotechnology and Laboratory Science, College of Medicine, Chang Gung University, Taoyuan City 33302, Taiwan

**Keywords:** 7-ketocholesterol, malonyl-CoA, mevalonic acid

## Abstract

The major oxidized product of cholesterol, 7-Ketocholesterol (7KCh), causes cellular oxidative damage. In the present study, we investigated the physiological responses of cardiomyocytes to 7KCh. A 7KCh treatment inhibited the growth of cardiac cells and their mitochondrial oxygen consumption. It was accompanied by a compensatory increase in mitochondrial mass and adaptive metabolic remodeling. The application of [U-^13^C] glucose labeling revealed an increased production of malonyl-CoA but a decreased formation of hydroxymethylglutaryl-coenzyme A (HMG-CoA) in the 7KCh-treated cells. The flux of the tricarboxylic acid (TCA) cycle decreased, while that of anaplerotic reaction increased, suggesting a net conversion of pyruvate to malonyl-CoA. The accumulation of malonyl-CoA inhibited the carnitine palmitoyltransferase-1 (CPT-1) activity, probably accounting for the 7-KCh-induced suppression of β-oxidation. We further examined the physiological roles of malonyl-CoA accumulation. Treatment with the inhibitor of malonyl-CoA decarboxylase, which increased the intracellular malonyl-CoA level, mitigated the growth inhibitory effect of 7KCh, whereas the treatment with the inhibitor of acetyl-CoA carboxylase, which reduced malonyl-CoA content, aggravated such a growth inhibitory effect. Knockout of malonyl-CoA decarboxylase gene (*Mlycd^−/−^*) alleviated the growth inhibitory effect of 7KCh. It was accompanied by improvement of the mitochondrial functions. These findings suggest that the formation of malonyl-CoA may represent a compensatory cytoprotective mechanism to sustain the growth of 7KCh-treated cells.

## 1. Introduction

High levels of 7-ketocholesterol (7KCh), an oxysterol derived from the oxidation of cholesterol (Chol), is detected in the vascular plaques of atherosclerosis patients and in the plasma of those at high risk of cardiovascular diseases [1]. It can be catabolized via the intra- and extrahepatic pathways [2,3,4]. The latter pathway involves sterol O-acyltransferase-mediated acylation and the reverse transport of the esterified form to high-density lipoprotein (HDL) [4]. A reduction of the sterol O-acyltransferase expression in heart leads to 7KCh accumulation and tissue damage. Highly elevated 7KCh levels were found in the red blood cells (RBCs) of heart failure patients [5]. It is envisaged that 7KCh can be an important risk factor for cardiovascular diseases and heart failure.

Heart tissue utilizes a number of energy sources for maintaining its continual contraction. The preference for fuel sources varies with developmental stages, shifting from glucose and lactate for fetal hearts to fatty acids for adult hearts. The remodeling of energy metabolism occurs in response to stressful conditions. Different physiological and pathophysiological conditions, including physical exercise, hypoxia, hypertrophy, and heart failure, causes the cardiac cells to switch to glucose for ATP production [6]. Altered energy metabolism in cardiac tissues is postulated to contribute to the pathogenesis of cardiovascular diseases such as cardiomyopathy and heart failure [7,8].

Exposure to 7KCh has an impact on redox homeostasis and cellular metabolism. It induces reactive oxygen species (ROS) generation in the endothelial cells and cardiomyocytes [5,9,10] and inflicts cellular damage. The other oxidized products of cholesterol cause ROS production, which is implicated in cognitive impairment and the development of cataracts [11,12]. Mitochondria represent an important endogenous source of ROS [13], which can damage the mitochondria in a reciprocal manner. Oxysterols and Chol accumulate in the mitochondria in the cardiac tissues of the animal models subject to ischemia reperfusion [14,15]. It is associated with anomalous changes in the mitochondria, such as the peroxidation of membrane lipids and the loss of the mitochondrial membrane potential [15]. Given the central roles of mitochondria in the metabolism, mitochondrial dysfunction is accompanied by the reprogramming of the metabolism in cardiomyocytes. We have recently reported that a 7KCh treatment induced changes in the cholesterol and lipid metabolism of HL-1 cells [16]. The genes involved in mevalonic acid biosynthesis and the metabolism of fatty acids, triacylglycerides and ketone bodies were down-regulated, while those involved in cholesterol transport and esterification were up-regulated [16]. Intriguingly, 7KCh enhanced the transcription of the genes regulated by ATF4, which has been recently identified as a key regulator of mitochondrial stress [17]. These findings have prompted us to study whether 7KCh induces mitochondrial stress and reprogramming of the cellular metabolism, in particular, energy metabolism.

In the present study, we demonstrate that 7KCh exposure induces the reprogramming of energy metabolism and mitochondrial dysfunction in cardiomyocytes and inhibits their growth. It is associated with a compensatory increase in the mitochondrial mass and malonyl-CoA accumulation. Malonyl-CoA suppresses carnitine palmitoyltransferase-1 (CPT-1) activity and β-oxidation. Studies involving the use of pharmaceutical inhibitors and *Mlycd^−/−^* knockout cells indicated that an increase in malonyl-CoA reverses the 7KCh-induced mitochondrial defects and growth inhibition.

## 2. Results

### 2.1. The Growth of Cardiomyocytes Is Inhbited by 7KCh

Decreases in the plasma HDL levels and increases in 7KCh in RBCs correlated with the prevalence of cardiovascular diseases and heart failure [5], implying that 7KCh may affect the physiology of cardiomyocytes. The treatment of cardiomyocytes resulted in the cellular uptake of 7KCh. The immunofluorescence assay with an anti-7KCh antibody was used to detect the localization of 7KCh in the HL-1 cells. The fluorescence of the antibody-stained 7KCh was significantly elevated in the 7KCh-treated HL-1 cells (Figure S1; lower left panel) compared with that of untreated cells (Figure S1; upper left panel), suggesting the uptake and intracellular accumulation of 7KCh.

**Figure 1:**
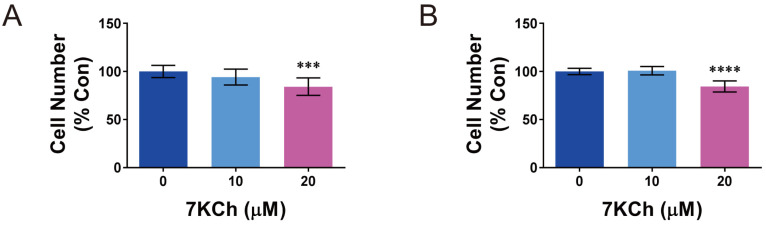
7KCh inhibits the growth of cardiomyocytes. HL-1 (**A**) or AC16 (**B**) cells were seeded at a cell density of 5 × 10^4^ per well of a 24 well culture plate, and after attachment, they were treated with the indicated concentrations of 7KCh for 24 h. Cells were stained with Hoechst 33342. The cell number was quantified using IN Cell Analyzer 1000 and is expressed as the percentage relative to the untreated cells. Data are mean ± SD of six experiments. *** *p* < 0.005, **** *p* < 0.001 vs. the 0 μM 7KCh treatment group.

We examined the effect of 7KCh on the growth of cardiac cell lines HL-1 and AC16. The HL-1 and AC16 cells were treated without or with 10 or 20 μM 7KCh. The concentrations used were in the physiological range of 7KCh. The erythrocytic 7KCh levels in the healthy volunteers were between 1 and 2 μM [5,18], and the blood 7KCh levels in the heart failure patients were at least 10-to 20-fold higher than those of the normal controls [5]. 7KCh inhibited growth of HL-1 and AC16 cells. The numbers of HL-1 and AC16 cells that were treated with 20 μM 7KCh were about 15% lower those of the untreated cells (Figure 1A,B).

### 2.2. Mitochondrial Dysfunction and ROS Formation in Cardiomyocytes Are Induced by 7KCh

**Figure 2:**
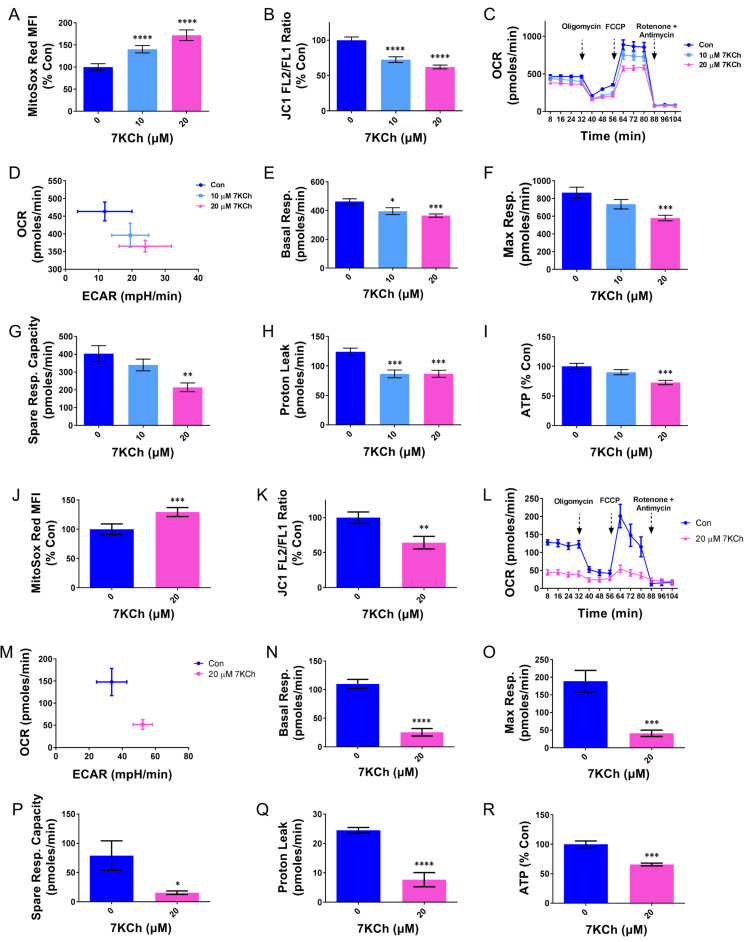
Mitochondrial dysfunction in cardiomyocytes is caused by 7KCh. HL-1 (**A**–**I**) and AC16 (**J**–**R**) cells were treated with the indicated concentrations of 7KCh for 24 h and stained with MitoSOX red (**A**,**J**) or JC1 (**B**,**K**). The mean fluorescence intensity (MFI) of the MitoSOX red-stained cells is expressed as the percentage relative to that of the untreated control (**A**,**J**). The ratio of the MFI of FL2 channel to that of FL1 channel (i.e., JC1 FL2/FL1 ratio) was calculated and is expressed as the percentage relative to that of the untreated control (**B**,**K**). Data are mean ± SD, N = 12. ** *p* < 0.01, *** *p* < 0.001, **** *p* < 0.005 vs. the 0 μM 7KCh treatment group. (**C**,**L**) The HL-1 cells or AC16 cells were treated with the indicated concentrations of 7KCh for 24 h and subjected to Seahorse respirometry analysis. Oligomycin, FCCP, rotenone, and antimycin were injected at appropriate timepoints. The oxygen consumption rate (OCR) and extracellular acidification rate (ECAR) were assessed. The OCR is normalized to cell number and plotted as a function of time of the 7KCh-treated cells (**C**,**L**). The baseline OCR and ECAR of the cells which were treated with the vehicle (*Con*) or with the indicated concentrations of 7KCh are normalized to cell number and plotted on the energy map (**D**,**M**). A representative of three experiments is shown. The basal respiration (**E**,**N**), maximum respiration (**F**,**O**), spare respiratory capacity (**G**,**P**), and proton leak (**H**,**Q**) were calculated. (**I**,**R**) Cells were similarly treated and extracted for ATP quantification. The ATP level is expressed as the percentage relative to that of the untreated control. Data are mean ± SD of six experiments. * *p* < 0.05, ** *p* < 0.01, *** *p* < 0.001, **** *p* < 0.005 vs. the 0 μM 7KCh treatment group.

Our previous study showed that 7KCh induces oxidative stress in cardiomyocytes [5]. Consistent with this, the HL-1 or AC16 cells were treated with 7KCh, stained with MitoSOX Red, and analyzed cytometrically. The fluorescence of MitoSOX Red was significantly elevated in the treated cells (Figure 2A,J), indicating that 7KCh may cause mitochondrial ROS generation. It is possible that 7KCh may induce the functional alteration of the mitochondria. The effect of 7KCh on the mitochondrial membrane potential ΔΨm was studied. The 7KCh-treated cells were stained with JC-1 dye and analyzed by flow cytometry. The mean fluorescence intensities (MFI) of channels FL2 and FL1 were measured, and their ratio (i.e., FL2 MFI/FL1 MFI), an indicator of ΔΨm, was calculated. The treatment of the HL-1 and AC16 cells with 20 μM 7KCh resulted in 40% decreases in ΔΨm (Figure 2B,K). To further delineate how 7KCH affects the mitochondrial respiration of cardiomyocytes, we resorted to mitochondrial respirometry to study such changes. The oxygen consumption rate (OCR) and extracellular acidification rate (ECAR) of the 7KCh-treated cells were assessed in real-time using a Seahorse XF24 Extracellular Flux Analyzer. The HL-1 cells that were treated without or with 10 or 20 μM 7KCh for 24 h had a reduced basal OCR, but an increased ECAR (Figure 2C–E). Such a shift in energy metabolism toward glycolysis was also observed in the AC16 cells treated with 20 μM 7KCh (Figure 2L–N). The use of respiration inhibitors (oligomycin, FCCP, rotenone, and antimycin A) allowed us to define the OCRs due to maximum respiration, the spare respiratory capacity, and proton leak. Both the maximum respiration and spare respiratory capacity of the HL-1 and AC16 cells diminished in response to 7KCh (Figure 2F,G,O,P). The proton leak was substantially reduced in the 7KCh-treated HL-1 and AC16 cells (Figure 2H,Q). Consistent with the reduction of respiratory function, the ATP levels in the HL-1 and AC16 cells treated with 20 μM 7KCh were about 30% and 35% lower than that of the control cells, respectively (Figure 2I,R). These findings suggest that 7KCh inhibits electron transport and oxidative phosphorylation and induces ROS generation.

### 2.3. Compensatory Biogenesis of Mitochondria in 7KCh-Treated Cardiomyocytes

**Figure 3:**
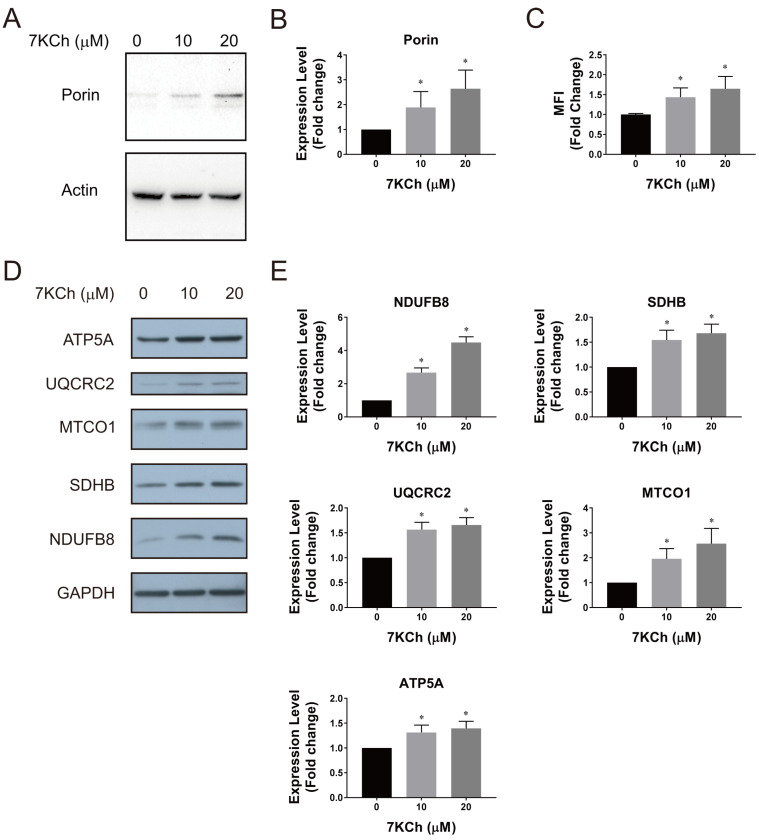
Compensatory mitochondrial biogenesis is induced by 7KCh. (**A**,**B**) HL-1 cells were treated with the indicated concentrations of 7KCh and analyzed for the expression of porin and actin by immunoblotting with respective antibodies. A representative of three experiments is shown. The band intensities of porin and actin were quantified. The band intensity of porin was normalized to that of actin and is expressed as the fold change relative to the 0 μM 7KCh treatment group. Data are mean ± SD of three experiments. * *p* < 0.05 vs. the 0 μM 7KCh treatment group. (**C**) Cells were similarly treated, and the mitochondrial mass was analyzed by MitoTracker Green staining and flow cytometric analysis. The MFI of the MitoTracker Green-stained cells is expressed as the fold change relative to that of the untreated control. Data are mean ± SD, N = 9. * *p* < 0.05 vs. the 0 μM 7KCh treatment group. (**D**,**E**) Expression of characteristic respiratory complex proteins. HL-1 cells were treated with the indicated concentrations of 7KCh, and analyzed for expression of NDUFB8, SDHB, UQCRC2, ATP5F1A, MTCO1, and glyceraldehyde-3-phosphate dehydrogenase (GAPDH). A representative of three experiments is shown. The band intensities of various proteins were quantified. The band intensities of these proteins are normalized to that of GAPDH, and expressed as the fold change relative to the 0 μM 7KCh treatment group. Data are mean ± SD of three experiments. * *p* < 0.05 vs. the 0 μM 7KCh treatment group.

The deteriorative changes in respiratory function are accompanied by an increase in the mitochondrial mass. As shown in Figure 3A,B, the porin level increased with the 7KCh concentration. In addition, the mitochondrial mass was evaluated by Mitotracker Green staining and cytometric analysis. The mean fluorescence intensity (MFI) of the stained HL-1 cells that had been treated with 20 μM 7KCh was 65% higher than that of the control cells (Figure 3C). These findings suggest that 7KCh causes mitochondrial biogenesis. Consistent with this, the levels of various mitochondrial respiratory chain proteins increased in the 7KCh-treated cells (Figure 3D). The nuclear gene-encoded complex I protein NADH:ubiquinone oxidoreductase subunit B8 (NDUFB8), complex II protein succinate dehydrogenase complex iron sulfur subunit B (SDHB), complex III protein ubiquinol-cytochrome C reductase core protein 2 (UQCRC2), and ATP Synthase F1 Subunit α (ATP5F1A), as well as mitochondrial gene-encoded complex IV protein cytochrome C oxidase I (MTCO1), increased in their expression, albeit to different extents, in the 7KCh-treated cells (Figure 3E). These findings suggest that 7KCh induces compensatory mitochondrial biogenesis in cardiac cells.

### 2.4. Energy Metabolic Profiling and the Changes in Metabolic Fluxes in the 7KCh-Treated Cardiomyocytes

**Figure 4:**
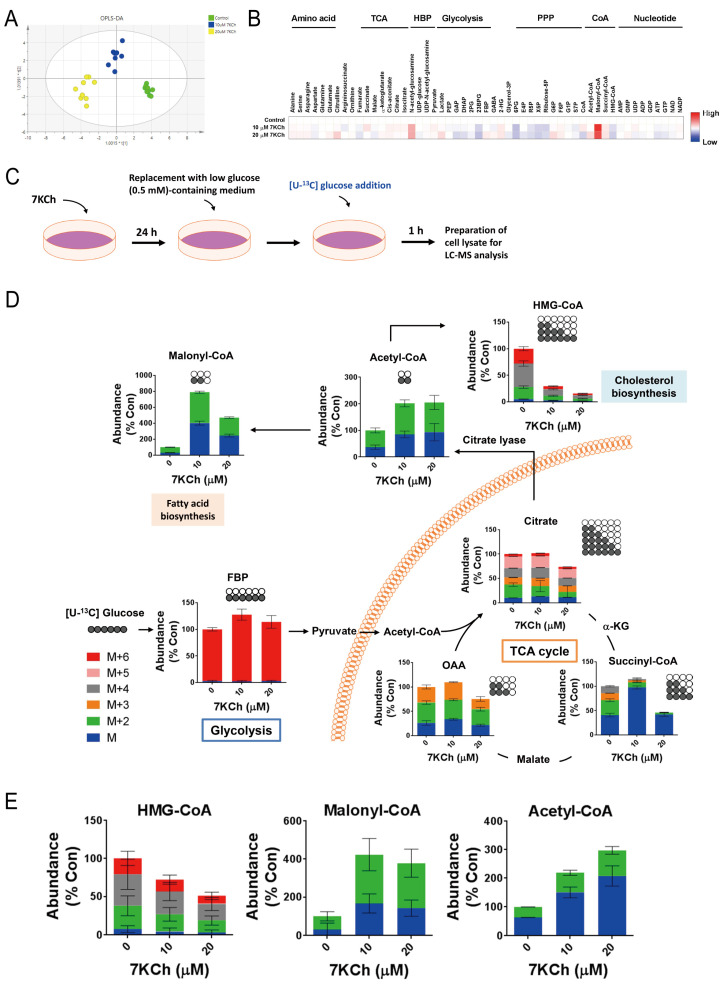
The reprogramming of cardiomyocytic metabolism and malonyl-CoA accumulation is induced by 7KCh. (**A**,**B**) The HL-1 cells were treated with the vehicle (Control) or with the indicated concentrations of 7KCh for 24 h, and subsequently, harvested for LC-MS/MS analysis in the electrospray ionization (ESI) negative ion mode. The OPLS-DA score plot (**A**) and the heatmap showing metabolites with significant differences between the 7KCh treatment group versus the control (**B**) are shown. The percent relative abundance is color-coded. Data are mean ± SD of six experiments. (**C**) A schematic diagram of the 7KCh treatment and [U-^13^C] glucose labeling procedure is shown. HL-1 (**D**) and AC16 (**E**) cells were treated with the vehicle or with 10 or 20 μM 7KCh for 24 h, labeled with [U-^13^C] glucose for 1 h, and harvested for MS and isotopologue analysis. Distribution of M (blue), M+2 (i.e., with 2 ^13^C atoms; green), M+3 (orange), M+4 (grey), M+5 (pink) and M+6 (red) isotopologues of metabolites were analyzed using the liquid chromatography/time-of-flight/mass spectrometry (LC-TOF-MS) system. The abundances of the selected metabolites (malonyl-CoA, acetyl-CoA, HMG-CoA, FBP, citrate, succinyl-CoA, and OAA) are expressed as the percentage relative to the 0 μM 7KCh treatment group (*Con*). The histograms are mapped onto the biochemical pathways (**D**). (**E**) The levels of HMG-CoA, malonyl-CoA and acetyl-CoA in AC16 cells treated with the indicated concentrations of 7KCh are shown. Data are mean ± SD of six experiments.

We applied a metabolomic approach for studying the metabolic reprogramming associated with the functional changes in the mitochondria. The metabolites involved in pathways such as glycolysis, pentose phosphate, and the citric acid cycle were analyzed by the liquid-chromatography coupled with tandem mass spectrometry (LC-MS/MS). A twenty-four hour treatment of HL-1 cells with 20 μM 7KCH caused a concentration-dependent change in the metabolome (Figure 4A). Increased levels of glucose-6-phosphate, fructose-1,6-bisphosphate (FBP), and lactate and decreased levels of 2-phosphoglycerate and 2,3-bisphosphoglycerate indicate an increase in the glycolytic rate (Figure 4B,D). Notably, malonyl-CoA accumulated at a high level in these cells. In contrast, the 3-hydroxy-3-methyl-glutaryl-coenzyme A (HMG-CoA) level decreased in the 7KCh-treated cells compared with that of the untreated cells. The accumulation of malonyl-CoA and the reduction of HMG-CoA formation were also observed in the 7KCh-treated AC16 cells (Figure 4E). We further examined the metabolism of malonyl-CoA using [U-^13^C] glucose labeling and tracking. The HL-1 cells were treated with 20 μM 7KCh and labeled with [U-^13^C] glucose. The relative abundances of the isotopologues of various metabolites were analyzed. As shown in Figure 4D,E, the M and M+2 isotopologues of malonyl-CoA increased, while the M, M+2, M+4, and M+6 isotopologues of HMG-CoA decreased. Interestingly, the total levels of the TCA cycle intermediates, citrate, succinyl-CoA, and oxaloacetate (OAA), remained unchanged or slightly increased in the cells treated with 10 μM 7KCh. However, these metabolites declined substantially in abundance upon exposure to the treatment with 20 μM 7KCh. An analysis of the isotopologues of the metabolites revealed that in addition to the M+2 and M+4 isotopologues formed from TCA cycle, the M+3 isotopologue of oxaloacetate generated in the anaplerotic reactions (Figure 4D). The proportions of different isotopologues of citrate were nearly unchanged in the cells treated with 20 μM 7KCh. In contrast, the proportions of isotopologues other than that of the M+2 succinyl-CoA decreased substantially. These findings suggest that the flux of the conversion of citrate to the downstream TCA intermediates is inhibited by 7KCh.

### 2.5. Fatty Acid Oxidation in Cardiomyocytes Is Inhibited by 7KCh

**Figure 5:**
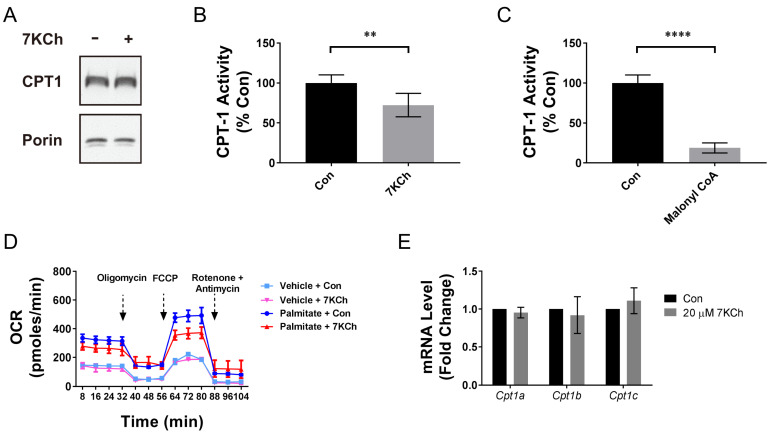
CPT-1 activity and β-oxidation in cardiomyocytes are inhibited by 7KCh. (**A**,**B**) The HL-1 cells were treated with the vehicle (Con) or 20 μM 7KCh for 24 h, and a crude mitochondrial fraction was prepared for the analysis of CPT-1 and porin expression by immunoblotting (**A**) and for the measurement of CPT-1 activity (**B**). For immunoblotting (**A**), a representative of three experiments is shown. (**B**) The CPT-1 activity of the 7KCh-treated cells is expressed as the percentage of that of untreated control cells. Data are mean ± SD, N = 6. ** *p* < 0.01 vs. the untreated control cells. (**C**) The crude mitochondrial fraction of the HL-1 cells was treated with the vehicle (Con) or 5 μM malonyl-CoA and assayed for the CPT-1 activity. Data are mean ± SD, N = 6. The CPT-1 activity of the malonyl-CoA treatment group is expressed as the percentage of that of Con group. **** *p* < 0.005 vs. the Con group. (**D**) The HL-1 cells were treated with the vehicle (Con) or 20 μM 7KCh for 24 h and were subjected to fatty acid oxidation assay using palmitate-BSA as a substrate (or BSA as the vehicle control for palmitate treatment; vehicle). A representative of three experiments is shown. (**E**) The cells were similarly treated, and the total RNA was extracted for the qRT-PCR-based quantification of *Cpt1a*, *Cpt1b*, and *Cpt1c* expression. Data are mean ± SD, N = 3.

The formation of malonyl-CoA has its functional implication. Malonyl-CoA is an inhibitor of CPT-1 involved in the fatty acid uptake into the mitochondria [19,20]. We isolated the mitochondria from the HL-1 cells treated without 7KCh and assayed the CPT-1 activity. The expression levels of CPT-1 were similar in the mitochondrial preparations to those of the control and treated cells (Figure 5A). The CPT-1 activity level was significantly lower in the mitochondria from the 7KCh-treated cells than it was in those from the control cells (Figure 5B). As control, malonyl-CoA directly inhibited the CPT-1 activity of the mitochondria (Figure 5C). These findings suggest that 7KCh inhibits the CPT-1 activity, and probably, β-oxidation in the cardiomyocytes.

To study the possibility that 7KCh inhibits the cardiomyocytic β-oxidation, we studied the fatty acid oxidation (FAO) in the HL-1 cells. The HL-1 cells were treated with or without 7KCh, and the palmitate (PA)-stimulated (or vehicle-stimulated) oxygen consumption was measured using the Seahorse XF24 Extracellular Flux Analyzer. The PA-stimulated oxygen consumption represents the reliance of the cells on β-oxidation for energy. 7KCh inhibited the basal respiration, maximum respiration, and spare respiratory capacity in the cells supplied with palmitate as an energy source (Figure 5D). In contrast, 7KCh did not significantly alter these respiration parameters in the cells the treated with the vehicle. The effect of 7KCh on PA-stimulated oxygen consumption is not attributable to expression of different CPT-1 isoforms. The transcripts of *Cpt1a*, *Cpt1b,* and *Cpt1c* genes, as quantified by RT-qPCR, did not differ between the 7KCh-treated and control cells (Figure 5E). These findings suggest that 7KCh may suppress the cardiomyocytic β-oxidation through the malonyl-CoA-mediated inhibition of CPT-1 activity (Figure 5A).

### 2.6. Inhibition of the Mevalonic Acid (MVA) Pathway Contributes to the Growth Retardation Effect of 7KCh

**Figure 6:**
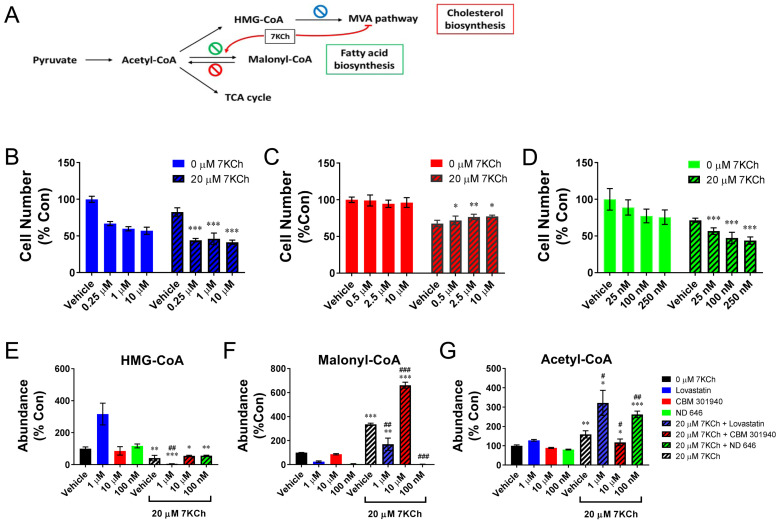
Accumulation of malonyl-CoA partially reverses the growth inhibitory effect of 7KCh. (**A**) A schematic diagram shows the enzymatic reactions at which pharmacological inhibitors act. CBM 301940 is an inhibitor of malonyl-CoA decarboxylase (red stop sign); ND 646 is an inhibitor of acetyl-CoA carboxylase (green stop sign); lovastatin is an inhibitor of HMGCR (blue stop sign). (**B**–**D**) The HL-1 cells were seeded at a cell density of 5 × 10^4^ per well of a 24-well culture plate, and after attachment, they were treated with the vehicle (vehicle) or with 0.25, 1, or 10 μM lovastatin (**B**), with the vehicle (vehicle) or with 0.5, 2.5, or 10 μM CBM 301940 (**C**), or with the vehicle (vehicle) or with 25, 100 or 250 nM ND 646 (**D**), in addition to 0 μM (i.e., the DMSO vehicle control; solid bar) or 20 μM 7KCh (striped bar) for 24 h. The cells were stained with Hoechst 33342, and the cell number was quantified using IN Cell Analyzer 1000. Data are mean ± SD of six experiments. * *p* < 0.05, ** *p* < 0.01, *** *p* < 0.005 vs. the vehicle group for the respective inhibitor treatment. (**E**–**G**) The cells were similarly treated with 7KCh and the pharmacological inhibitors and extracted for the quantification of HMG-CoA, malonyl-CoA, and acetyl-CoA. The abundances of these metabolites are expressed as the percentage relative to those of the cells that were treated with neither 7KCh nor any pharmacological inhibitor. Data are mean ± SD, N = 3. * *p* < 0.05, ** *p* < 0.01, *** *p* < 0.005, the 7KCh treatment group vs. the non-treatment group; # *p* < 0.05, ## *p* < 0.01, ### *p* < 0.005 vs. the vehicle group for the respective inhibitor treatment.

The reduction of the HMG-CoA level in the 7KCh-treated HL-1 cells suggests that the inhibition of the MVA pathways may retard the growth of cardiac cells. To study this possibility, we treated the HL-1 cells with 0.25, 1, and 10 μM lovastatin, in addition to 20 μM 7KCh, and determined the cell number. Lovastatin is an inhibitor of enzyme 3-hydroxy-3-methylglutaryl-coenzyme A reductase (HMGCR) (Figure 6A) [21]. The treatment with 1 μM lovastatin alone caused a three-fold increase in the HMG-CoA level, and a 40% decrease in the cell number (Figure 6B). The co-treatment with 7KCh and lovastatin reduced the HMG-CoA level by 94%. It is consistent with our previous finding that 7KCh represses the expression of *Acat2* and *Hmgcs1* genes [16], the products of which act upstream of HMGCR in the MVA pathway. Such a co-treatment caused a 54% decrease in the cell number (Figure 6B). These findings suggest that the products of the MVA pathway, such as coenzyme Q (CoQ), may play essential roles in growth of cardiomyocytes. It is in agreement with a decline in the CoQ_10_ level in the 7KCh-treated cells (Figure S2).

### 2.7. Malonyl-CoA Production Is Cytoprotective

To study the physiological roles of malonyl-CoA accumulation in the 7KCh-treated cells, we studied the effect of the inhibitors of malonyl-CoA decarboxylase and acetyl-CoA carboxylase on cell growth (Figure 6A). Malonyl-CoA decarboxylase (MLYCD) catalyzes the degradation of malonyl-CoA to acetyl-CoA; acetyl-CoA carboxylase catalyzes the carboxylation of acetyl-CoA to form malonyl-CoA. The HL-1 cells were co-treated with 20 μM 7KCh and 0.5, 2.5, or 10 μM malonyl-CoA decarboxylase inhibitor CBM 301940, and the cell number was determined. The CBM 301940 treatment, which significantly increased the intracellular ratio of malonyl-CoA level to acetyl-CoA, alleviated the growth retardation effect of 7KCh (Figure 6C,F). For the study with acetyl-CoA carboxylase inhibitor, the HL-1 cells were co-treated with 25,100,250 nM ND 646, and the cell number was determined. The ND 646 treatment, which suppressed the intracellular formation of malonyl-CoA, acted synergistically with 7KCh to inhibit cell growth (Figure 6D,G). These findings suggest that the formation of malonyl-CoA is beneficial to the growth of cardiomyocytes and may represent a compensatory cytoprotective response to 7KCh.

**Figure 7:**
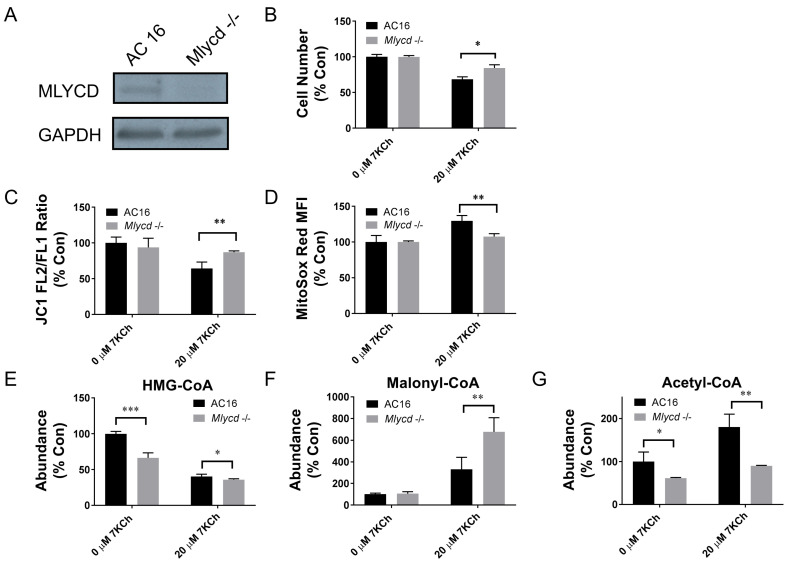
Knockout of the *Mlycd* gene restores mitochondrial functions and counteracts the growth inhibitory effect of 7KCh. (**A**) The *Mlycd^−/−^* and control AC16 cells were analyzed for the expression of MLYCD and GAPDH by immunoblotting. A representative of three experiments is shown. (**B**) The *Mlycd^−/−^* and control AC16 cells were treated with 0 (i.e., the vehicle control) or 20 μM 7KCh for 24 h and stained with Hoechst 33342. The cell number was quantified using IN Cell Analyzer 1000 and is expressed as the percentage relative to the untreated AC16 cells. Data are mean ± SD of six experiments. * *p* < 0.05 vs. AC16 cells. (**C**,**D**) The *Mlycd^−/−^* and control AC16 cells were treated with the indicated concentrations of 7KCh for 24 h and stained with JC1 (**C**) or MitoSOX red (**D**). The ratio of the MFI of FL2 channel to that of FL1 channel (i.e., JC1 FL2/FL1 ratio) was calculated and is expressed as the percentage relative to that of the untreated AC16 cells. The MFI of the MitoSOX red-stained cells is expressed as the percentage relative to that of the untreated AC16 cells. Data are mean ± SD, N = 6. ** *p* < 0.01 vs. AC16 cells. (**E**–**G**) The *Mlycd^−/−^* and control AC16 cells were similarly treated with 7KCh and extracted for the quantification of HMG-CoA, malonyl-CoA, and acetyl-CoA. The abundances of these metabolites are expressed as the percentage relative to those of the untreated AC16 cells. Data are mean ± SD, N = 6. * *p* < 0.05, ** *p* < 0.01, *** *p* < 0.005 vs. AC16 cells.

To study if malonyl-Co-A accumulation in the cardiomyocytes offers a cytoprotective effect, we derived *Mlycd^−/−^* cells from AC16 cells using the CRISPR/Cas9 technology. The expression of the MLYCD protein was nullified in the *Mlycd^−^*^/*−*^ cells (Figure 7A). *Mlycd* knockout mitigated the growth inhibitory effect of 7KCh (Figure 7B). It was associated with the restoration of ΔΨm and decreased mitochondrial ROS production (Figure 7C,D). The knockout of the *Mlycd* gene maintained the steady state level of malonyl-CoA, while it reduced that of acetyl-CoA (Figure 7F,G). This increased the malonyl-CoA/acetyl-CoA ratio by about 70%. The malonyl-CoA/acetyl-CoA ratio was elevated four-fold in the 7KCh-treated *Mlycd^−/−^* cells as compared to that of the treated AC16 cells (Figure 7F,G). These findings validate that an increase in malonyl-CoA production can relieve the cardiomyocytes from the 7KCh-induced growth inhibition and mitochondrial dysfunction.

### 2.8. The Expression of the Genes Involved in Malonyl-CoA Metabolism in Cardiomyocytes Is Differentially Modulated by 7KCh

**Figure 8:**
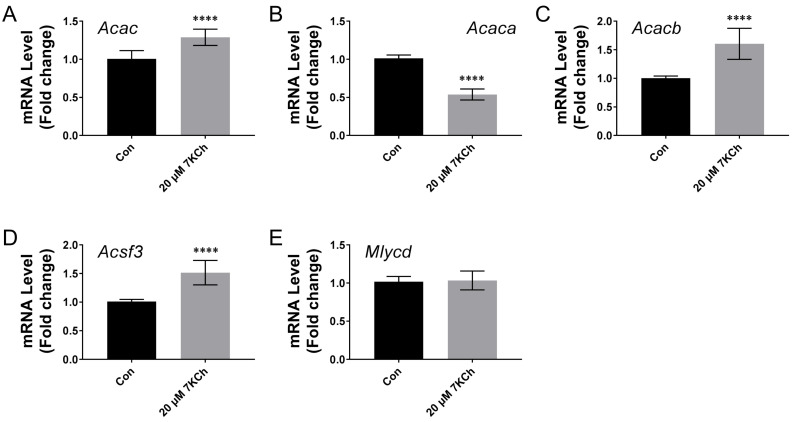
The transcription of the genes encoding malonyl-CoA metabolizing enzymes is regulated by 7KCh. The HL-1 cells were treated with 0 (i.e., the vehicle control; *Con*) or 20 μM 7KCh, and the total RNA was extracted for the qRT-PCR-based quantification of expression of the *Acac* (**A**) (i.e., all transcript variants encoding different acetyl-CoA carboxylase isoforms), *Acaca* (**B**), *Acacb* (**C**), *Acsf3* (**D**), and *Mlycd* (**E**) genes. The abundances of these metabolites are expressed as the fold change relative to the untreated control. Data are mean ± SD, N = 12. **** *p* < 0.001 vs. the untreated control.

We studied the effect of 7KCh on the expression of the genes encoding acetyl-CoA carboxylase (ACAC) and acyl-CoA synthetase family member 3 (ACSF3). The level of the acetyl-CoA carboxylase gene transcript, as measured by RT-qPCR using ACAC universal primers, was elevated in the 7KCh-treated HL-1 cells (Figure 8A). There are two ACAC isoforms, namely acetyl-CoA carboxylase α (ACACA) and β (ACACB), in mammals [22]. The *Acacb* transcript level increased, while that of the *Acaca* transcript declined (Figure 8B). In addition, there was a significant increase in the *Acsf3* transcript level (Figure 8D). In contrast, the *Mlycd* transcript level remained nearly the same after the 7KCh treatment. These findings suggest that 7KCh enhances the expression of *Acacb* and *Acsf3* genes.

## 3. Discussion

It is possible that the 7KCh-induced disruption of normal energy metabolism and metabolic reprogramming in the cardiomyocytes may play important pathogenic roles in cardiovascular diseases. The way in which 7KCh induces metabolic changes in cardiomyocytes remains elusive. In the present work, we demonstrate that 7KCh causes mitochondrial dysfunction, ROS production, and growth retardation. Malonyl-CoA accrues in the cardiomyocytes in response to 7KCh and inhibits CPT-1 activity and β-oxidation. The studies involving pharmacological inhibitors and *Mlycd*-knockout cardiomyocytes indicate that an experimental rise in the malonyl-CoA level restores the mitochondrial ΔΨm and redox homeostasis and reverses the growth inhibitory effect of 7KCh.

It is speculative whether 7KCh directly affects the cardiomyocytic mitochondrial functions. The treatment of the translocator protein ligand 4′-chlorodiazepam suppressed the formation of oxysterols and partially restored the mitochondrial respiration [15], implying an adverse effect of 7KCh on the mitochondria. 7KCh damages the mitochondrial DNA and causes a loss of ΔΨm in retinal pigment cells [23]. We found that the 7KCh treatment resulted in reduction of ΔΨm in the HL-1 and AC16 cells, which were treated with 20 μM KCh (Figure 2B,K). It was associated with the decreases in basal respiration, maximum respiration, as well as spare respiratory capacity in these cells (Figure 2E,F,G,N,O,P). Both the flux of mitochondrial metabolism and the efficiency of respiratory processes, but not the expression of mitochondrial proteins, are adversely affected by 7KCh. The expression of the typical respiratory complex proteins actually increased after the 7KCh treatment (Figure 3D,E). The flux of the TCA cycle decreases in the treated cells (Figure 4), contributing to the decline in oxygen consumption. The efficiency of electron transport is lowered by 7KCh, which is indicated by an increase in ROS generation in the treated cells (Figure 2A,J). The proper assembly of the respiratory supercomplex is essential to efficient electron transport. It has been found that the basal and maximum respiration and the spare respiratory capacity are reduced in the *PHB*-deficient cells, with impaired organization of the respiratory supercomplexes [24,25]. Consistent with such a notion, our previous study has shown that the *Phb1*, *Phb2*, *Higd1a*, and *Higd2a* transcript levels declined in the 7KCh-treated cells [16]. Prohibin 1 (PHB1) and 2 (PHB2) participate in the assembly of respiratory supercomplexes and the maintenance of their stability [26]. HIGD1A and HIGD2A are involved in the dynamic assembly of complexes III and IV and in supercomplex formation [27]. Moreover, changes in the anabolic pathways may hamper the electron transport. The reduction of the flux of the MVA pathway dwindled the supply of HMG-CoA (Figure 4B,D,E), which is the precursor of CoQ biosynthesis. As CoQs serve as an important electron carrier, their decrease is likely to impair the normal functioning of the electron transport chain (Figure S2).

The 7KCh-induced changes in the mitochondrial functions correlate with the reprogramming of cardiomyocytic energy metabolism. Increases in the levels of glucose-6-phosphate, fructose-1,6-bisphosphate, and lactate (Figure 4A), together with an increase in ECAR (Figure 2), indicate an increase in the glycolytic rate in the 7KCh-treated cardiac cells. Substantial decreases in TCA intermediates, such as citrate, succinyl-CoA, and oxaloacetate, in the cells treated with 20 μM 7KCh are suggestive of a reduction of the TCA cycle flux in these cells (Figure 4D). The isotopologue analysis reveals that the proportions of the M+2 and M+4 isotopologues of succinyl-CoA were similar to those of the corresponding isotopologues of citrate in the control cells. However, the levels of the M+2 and M+4 isotopologues of succinyl-CoA were substantially reduced by the 7KCh treatment. It is likely that the flux of the onward reaction of citrate in TCA cycle decreased upon 7KCh treatment. It also implies that citrate is probably involved in to reactions such as that catalyzed by citrate lyase. Interestingly, most of succinyl-CoA molecules remained unlabeled. These molecules formed from the unlabeled α-ketoglutarate, which may be derived from glutamate in a glutamate dehydrogenase-catalyzed reaction. Additionally, anaplerotic reactions made significant contribution to the oxaloacetate pool in the control and 7KCh-treated cells. Overall, pyruvate derived from glycolysis is converted either to lactate or to oxaloacetate (through anaplerotic reactions). Citrate synthase converts oxaloacetate to citrate, which can be metabolized in the TCA cycle or converted to acetyl-CoA for malonyl-CoA synthesis. In the 7KCh-treated cells, glycolysis is enhanced, while the TCA cycle is inhibited. More citrate molecules contribute to malonyl-CoA synthesis.

Malonyl-CoA itself is an inhibitor of CPT-1, which is involved in the fatty acid uptake into the mitochondria and β-oxidation (Figure 5C) [19]. The accumulation of malonyl-CoA in the 7KCh-treated cells reduced the use of fatty acids as fuel molecules for β-oxidation. Indeed, 7KCh inhibited the CPT-1 activity (Figure 5B) and palmitate-stimulated respiration (Figure 5D). It is evident that malonyl-CoA is cardioprotective. The pharmacological inhibition of MLYCD activity and the knockdown of the *Mlycd* gene improve the biomechanical functions of the post-ischemic heart [28,29]. The 7KCh-induced malonyl-CoA accumulation probably represents a compensatory mechanism to reduce the toxic effect of 7KCh. The pharmacological treatment with CBM 301940, which increased the intracellular malonyl-CoA level, lessened the growth inhibitory effect of 7KCh (Figure 6C,F). The growth of *Mlycd^−/−^* cardiac cells were inhibited to a much lesser extent than the control cells were after 7KCh exposure (Figure 7B). Such a cytoprotective effect was associated with the restoration of ΔΨm and a decrease in mitochondrial ROS generation, suggesting an improvement of the mitochondrial functions. These findings advocate that metabolic reprogramming occurs in the 7KCh-treated cardiomyocytes to compensate for mitochondrial dysfunction and growth defects.

An additional point about the malonyl CoA-inhibited β-oxidation is noteworthy. Our previous study has revealed that fatty acids can be released from phosphatidylcholines by phospholipase A2, and are used for esterification [16]. The fatty acid molecules are spared from β-oxidation through the action of malonyl-CoA. Malonyl-CoA appears not to serve as a precursor of fatty acid synthesis. The expression of the genes involved in fatty acid synthesis, such as fatty acid synthase and desaturases, are down-regulated [16].

The shift in energy metabolism from oxidative phosphorylation to glycolysis is physiologically relevant. Fatty acids are the major fuel for healthy adult hearts, responsible for 60–80% of ATP generation [30]. The remaining ATP molecules are derived from the metabolism of glucose and lactate. A change in fuel preference and energy metabolism may occur under different physiological or pathophysiological conditions. For instance, an increased glycolysis level was observed in the cardiac tissues isolated from the animal models of left ventricular hypertrophy [31,32,33]. The uptake of 2-deoxy-2-[^18^F] fluoro-D-glucose increased in the right ventricles of pulmonary arterial hypertension patients, and the uptake value correlated with the disease severity scores [34]. Additionally, it has been recently shown that myocardial infarction or pressure loading induces the cycling of specialized cardiomyocytes that may be involved in cardiac repair [35]. The activation of glycolysis is apparently essential to cardiomyocytic proliferation after an injury [36,37]. It is possible that the remodeling of energy metabolism in cardiomyocytes may affect cellular proliferation and tissue regeneration under certain circumstances, for example, the presence of high plasma 7KCh levels [38].

It is not unprecedented that the compensatory mitochondrial biogenesis occurs in response to mitochondrial dysfunction. Knockout of the leucine-rich pentatricopeptide repeat containing protein (LRPPRC)-encoding gene, whose mutations have been identified in Leigh syndrome, causes the defective assembly of the electron transport chain and triggers compensatory mitochondrial biogenesis [39]. Compensatory mitochondrial biogenesis occurs in cells with mitochondrial DNA mutations [40]. The carriers of LHON mutations display increases in the mitochondrial mass [41]. The declines in mitochondrial functions and ATP supply probably induce the expression of respiratory complex proteins and mitochondrial biogenesis in the 7KCh-treated cells (Figure 3). This is consistent with increases in the transcripts of the *Tfam*, *Tfb1m*, *Tfb2m*, and *Pprc1* genes [16], which are involved in mitochondrial transcription, ribosome assembly, and biogenesis [42,43].

The transcription of the genes encoding the malonyl-CoA-metabolizing enzymes is differentially modulated by 7KCh. The *Acacb* transcription is up-regulated at the expense of the transcription of the *Acaca* gene (Figure 8B,C), resulting in an overall increase in the ACAC-encoding transcript (Figure 8A). The ACACB protein, located at the outer mitochondrial membrane, promotes the formation of malonyl-CoA, which allosterically inhibits CPT-1 activity [22,44]. Such a change in the expression of ACAC isoforms in the 7KCh-treated cells is congruous with the inhibition of β-oxidation. ACSF3 is involved in the detoxification of malonate, which is a competitive inhibitor of succinate dehydrogenase [45]. The ACSF3-derived malonyl-CoA is used in the malonylation of proteins, which may be involved in the post-translational regulation of mitochondrial proteins and metabolism [45]. The knockout of the *Acsf3* gene in HEK 293T cells was found to alter their metabolism [46]. The enhanced transcription of the *Acsf3* gene in the 7KCh-treated cells may imply the role of protein malonylation in the observed metabolic changes.

The present study has limitations, such as cardiac cell lines were used and more work with animal models is needed to fully understand the cytoprotective mechanism. The results might be subject to technical variation caused by the methods used (e.g., cell counting).

## 4. Materials and Methods

### 4.1. Materials

Unless otherwise stated, all the chemicals were purchased from Sigma-Aldrich (St. Louis, MO, USA). We dissolved 7-Ketocholesterol (7KCh; Sigma-Aldrich) in dimethyl sulfoxide (DMSO). In most of the experiments, 7KCh was used at a concentration of 10 or 20 μM. Lovastatin (Caymen Chemical, Ann Arbor, MI, USA), CBM 301940 (Torcis Bioscience, Bio-Techne, Minneapolis, MN, USA), and ND 646 (Caymen Chemical) were dissolved in DMSO. Lovastatin was used at the concentration range 0.25–10 μM; CBM 301940 was used at the concentration range 0.5–10 μM; ND 646 was used at the concentration range 25–250 nM.

### 4.2. Cell Culture and Viability Assay

HL-1 atrial myocytes (Research Resource Identifier (RRID): CVCL_0303) were cultured in the fibronectin-gelatin-coated flasks containing the Claycomb medium (51800C, Sigma-Aldrich), which was supplemented with 10% HL-1 qualified fetal bovine serum (FBS; TMS-016, Sigma-Aldrich), 100 U/mL penicillin, 100 μg/mL streptomycin, 2 mM L-glutamine, and 0.1 mM norepinephrine in a humidified atmosphere of 5% CO_2_ at 37 °C, as previously described [47]. The AC16 human cardiomyocyte cell line (RRID: CVCL_4U18; EMD Millipore Corp., Temecula, CA, USA) was cultured in Dulbecco’s modified Eagle medium/nutrient mixture F-12 medium (DMEM/F-12; Thermo Fisher Scientific, Waltham, MA, USA) containing 12.5% FBS according to manufacturer’s instructions. The malonyl-CoA decarboxylase (*Mlycd*) gene knockout AC16 cells (i.e., *Mlycd^−/−^* cells) were generated using the CRISPR/Cas9 knockout service provided by the RNA Technology Platform and Gene Manipulation Core (National Core Facility for Biopharmaceuticals, Taipei, Taiwan).

For determination of the growth curves of the 7KCh-treated cells, 5 × 10^4^ cells were seeded in 12-well culture plate and treated with the indicated 7KCh concentrations (i.e., the concentrations indicated in the legends of the respective figures) for various periods. Cardiac cells were fixed in 3.7% formaldehyde for 10 min, and then stained with 5 μg/mL of Hoechst 33342 for 15 min. The cell number was determined using IN Cell Analyzer 1000 (GE Healthcare Life Sciences, Chicago, IL, USA) [48]. As a control for the 7KCh treatment, the cells were treated with the dimethyl sulfoxide (DMSO) vehicle. DMSO also served as the vehicle for lovastatin, CBM 301940, and ND 646.

### 4.3. Assessment of Mitochondrial Function Assays

#### 4.3.1. Oxygen Consumption Rate (OCR) and Extracellular Acidification Rate (ECAR) Determination and Mitochondrial Function Tests

The measurement of the OCR and ECAR was performed using Seahorse XF24-3 Analyzer (Agilent Technologies, Santa Clara, CA, USA) as previously described [49]. In brief, 8 × 10^3^ cells were seeded per well in an XF24 cell culture microplate and maintained in DMEM (Thermo Fisher Scientific). They were treated with the indicated concentrations of 7KCh. After 24 h, the medium was replaced with DMEM without sodium bicarbonate. The XF24 assay cartridge was prepared, loaded with 10 μM oligomycin, 3 μM FCCP, 10 μM antimycin A, and rotenone in the injection ports, and calibrated according to the manufacturer’s instruction. The oxygen consumption of the cells was measured using Seahorse XF-24 analyzer (Agilent Technologies) under basal condition and after the sequential injection of oligomycin (32 min), carbonyl cyanide 4-(trifluoromethoxy)phenylhydrazone (FCCP) (56 min), and antimycin A/rotenone (88 min). The proton flux in the medium was also monitored. The OCR and ECAR were automatically calculated.

Superoxide anion production was determined by MitoSOX Red staining and flow cytometry as previously described [50]. The mitochondrial mass and mitochondrial membrane potential were determined, respectively, by staining with MitoTracker Green and JC-1 and performing cytometric analyses as previously described [51,52]. BD LSR II flow cytometer (Becton Dickinson, Franklin Lakes, NJ, USA) was used for the analyses.

#### 4.3.2. Fatty Acid Oxidation (FAO) Assay

At 24 h before the experiment, 8 × 10^3^ cells were cultured per well in an XF-24 plate. The cells were treated with the indicated concentrations of 7KCh for 24 h. The medium was replaced with 375 μL of FAO Assay Medium (111 mM NaCl, 4.7 mM KCl, 1.25 mM CaCl_2_, 2 mM MgSO_4_, 1.2 mM NaH_2_PO_4_ supplemented with 2.5 mM glucose, 0.5 mM carnitine, and 5 mM HEPES, pH 7.4) and incubated at 37 °C for 45 min. Before the analysis, 87.5 μL of 1 mM palmitate–1% BSA conjugate or 1% BSA were added to each well. During the experiment, respiration was measured under basal condition and after the sequential injection of oligomycin, FCCP, and antimycin A/rotenone as described in Section 4.3.1.

### 4.4. Metabolite Analysis Using Liquid-Chromatography Coupled with Tandem Mass Spectrometry (LC-MS/MS)

The metabolites were extracted as previously described [53,54]. Briefly, the cells were treated with the indicated concentrations of 7KCh for 24 h, and then washed twice with cold phosphate-buffered saline (PBS). The metabolites were extracted with 80% (*v*/*v*) MeOH/H_2_O pre-equilibrated at 80 °C. The extract was collected into 1.5 mL tubes, vortexed for 5 min, and centrifuged at 12,000× *g* for 30 min. The resulting supernatant was dried using a centrifugal evaporator under reduced pressure. The samples were re-suspended in 200 μL of 1% acetic acid for mass spectrometric analysis, and then were analyzed using the Xevo TQ-XS Triple Quadrupole Mass Spectrometry System (Waters Corp., Milford, MA, USA) [16]. The chromatographic separation was achieved on a BEH C18 (100 × 2.1 mm, particle size of 1.7 µm; Waters Corp.) at 45 °C. The mobile phase consisted of eluent A (10 mM tributylamine (TBA)/15 mM acetic acid) and eluent B (10 mM TBA/15 mM acetic acid/50% ACN). The flow rate was set at 0.3 mL/min. The elution profile was as follows: 4% B, 6 min; linear gradient 4–50% B, 0.1 min; 50–60% B, 2.9 min; 60–100% B, 0.8 min, and 100% B for an additional 2.2 min. The mass spectrometer was operated in negative ion mode at an ESI voltage of 3 kV. The metabolites were analyzed using MassLynx software (v4.1; Waters Corp., Milford, MA, USA).

### 4.5. [U-^13^C] Glucose Labeling and Isotopologue Analysis

Stable isotope-labeling was performed as previously described with slight modifications [55]. The cells were treated with or without 7KCh for 24 h, and then incubated in the medium containing 0.5 mM glucose (i.e., low-glucose medium) for 0.5 h. [U-^13^C] Glucose was added to a final concentration of 20 mM and the incubation continued for 1 h. Metabolites were extracted in the ice-cold 80% methanol and analyzed using the Vion IMS QTOF Acquity UPLC system (Waters Corp.). Chromatographic separation was achieved on a BEH C18 (100 × 2.1 mm, particle size of 1.7 um; Waters Corp.) at 45 °C. The mobile phase consisted of eluent A (10 mM TBA/15 mM acetic acid) and eluent B (10 mM TBA/15 mM acetic acid/50% ACN). The flow rate was set at 0.3 mL/min. The elution profile was the same as that which is described in the preceding section. The mass spectrometer was operated in negative ion mode at an ESI voltage of 2.5 kV. Metabolites were analyzed using UNIFI software (v1.0.6171; Waters Corp.).

### 4.6. Isolation of Mitochondria and CPT-1 Activity Assay

Isolation of the mitochondria was performed by a modification of previously described method [56]. Around 1.5 × 10^6^ HL-1 cells were harvested and resuspended in ice-cold mitochondria isolation buffer (20 mM HEPES, 5 mM KH_2_PO_4_, 50 μM MgCl_2_, 250 mM sucrose, and 0.2% BSA, pH 7.5). The sample was homogenized with 20 passages using a 27 gauge needle. The homogenate was centrifuged at 500× *g* for 10 min, and the supernatant was retained. The pellet was washed with the mitochondria isolation buffer, and then subjected to centrifugation. The supernatant fractions were combined and centrifuged at 10,000× *g* for 30 min at 4 °C to obtain a crude mitochondrial pellet.

The CPT-1 activity was measured as described elsewhere with modifications [57]. The enriched mitochondria were suspended in 80 μL of mitochondria isolation buffer and incubated at 37 °C for 2 min. The enzymatic reaction was initiated by addition of 10 μL 1 mM palmitoyl-CoA and 10 μL 10 mM carnitine and incubated at 37 °C for 5 min. The product palmitoylcarnitine was analyzed using LC-MS under conditions that had been described previously [57].

### 4.7. Western Blotting and Immunofluorescence

The cells were rinsed with cold PBS, scraped, and collected for centrifugation. They were immediately lysed in a lysis buffer (20 mM Tris·HCl (pH 8), 1% Triton X-100, 137 mM NaCl, 1.5 mM MgCl_2_, 10% glycerol, 1 mM EGTA, 50 mM NaF, 1 mM Na_3_VO_4_, 10 mM β-glycerophosphate, 1 mM PMSF, 1 μg/mL leupeptin, and1 μg/mL aprotinin). The protein concentration of the lysate was determined using the Bradford method. The sample was analyzed by SDS-PAGE and immunoblotting with primary antibodies (including anti-VDAC1/porin antibody (ab14734; Abcam, Cambridge, UK), anti-actin (clone AC-40; Sigma-Aldrich), total OXPHOS rodent WB antibody cocktail (ab110413 (MS-604), Abcam), anti-CPT1A antibody (15184-1-AP; Proteintech group Inc., Rosemont, IL, USA), and anti-GAPDH antibody (GTX100118; GeneTex Inc., Irvine, CA, USA)), and appropriate secondary antibodies (including the horseradish-peroxidase (HRP)-conjugated goat anti-mouse antibody (sc-2005; Santa Cruz, Dallas, TX, USA), and the HRP-conjugated mouse anti-rabbit antibody (SC-2357; Santa Cruz Biotechnology, Dallas, TX, USA)).

For immunofluorescence staining, the cells were cultured in a 35 mm glass-bottomed culture dish (Mattek Life Sciences, Ashland, MA, USA), and after the 7KCh treatment, the cells were fixed in 4% paraformaldehyde/PBS for 1 h. The fixed cells were rinsed with PBS and stained with 1 μg/mL anti-7-ketochosterol antibody (MKC-100n; Japan Institute for the Control of Aging (JaICA) Nikken Seil Co., Ltd., Shizuoka, Japan) in PBS/0.1% Triton X-100/1% bovine serum albumin (BSA) at 4 °C overnight. After rinsing it with PBS, the sample was stained with 2 μg/mL anti-mouse DyLight 488-conjugated secondary antibody (Thermo Fisher Scientific) and counterstained with Hoechst 33342 at room temperature for 2 h. The sample was examined under a Zeiss LSM780 fluorescence microscope (Carl Zeiss Microscopy, Oberkochen, Germany).

### 4.8. Reverse Transcription-Quantitative Polymerase Chain Reaction (RT-qPCR)

The HL-1 cells were treated with 0, 10, 20, or 50 μM 7KCh for 24 h. They were washed with PBS, and subsequently, lysed with TRIzol reagent (Life Technologies, Carlsbad, CA, USA) for total RNA isolation. The total RNA was quantified using NanoDrop (Implen, Munich, Germany). The cDNA was synthesized from the total RNA using a RevertAid first-strand cDNA synthesis kit (catalog no. K1622; Thermo Fisher Scientific) according to the manufacturer’s protocol. The cDNA sample was mixed with the SsoFast EvaGreen supermix (catalog no. 172-5201; Bio-Rad, Hercules, CA, USA) in a reaction volume of 10 μL. PCR was performed with the CFX96 Touch real-time PCR detection system (Bio-Rad, Hercules, CA, USA). The relative expression of mRNAs was calculated using the comparative CT method and normalized to GAPDH expression. The primer sequences are listed in Table S1.

### 4.9. Statistical Analyses

The number of independent experiments (each with a triplicate set of samples) is stated or given as the N value in the respective figure legend. Data are mean ± SD. All the statistical analyses were performed with IBM SPSS 20.0 (IBM, Armonk, NY, USA). Two-way analysis of variance (ANOVA) with Sidak’s multiple comparison test, Mann–Whitney test, Kruskal–Wallis test with Dunn’s multiple comparison test, and Student’s *t* test were used where appropriate. A *p* value of < 0.05 is considered to be significant.

## Data Availability

The data presented in this study are available upon request from the corresponding author. The data are not publicly available due to patent application request.

## Supplementary Materials

The following supporting information can be downloaded at: https://www.mdpi.com/article/10.3390/ijms24054418/s1.
