# Supplementary material for: Malonyl-CoA Accumulation as a Compensatory Cytoprotective Mechanism in Cardiac Cells in Response to 7-Ketocholesterol-Induced Growth Retardation
Source: Int J Mol Sci. 2023 Feb 23;24(5):4418. doi: 10.3390/ijms24054418 (PMC10002498; doi:10.3390/ijms24054418)
Supplement: Supplementary file 1 [file ijms-24-04418-s001.zip › ijms-2191418-supplementary.pdf]

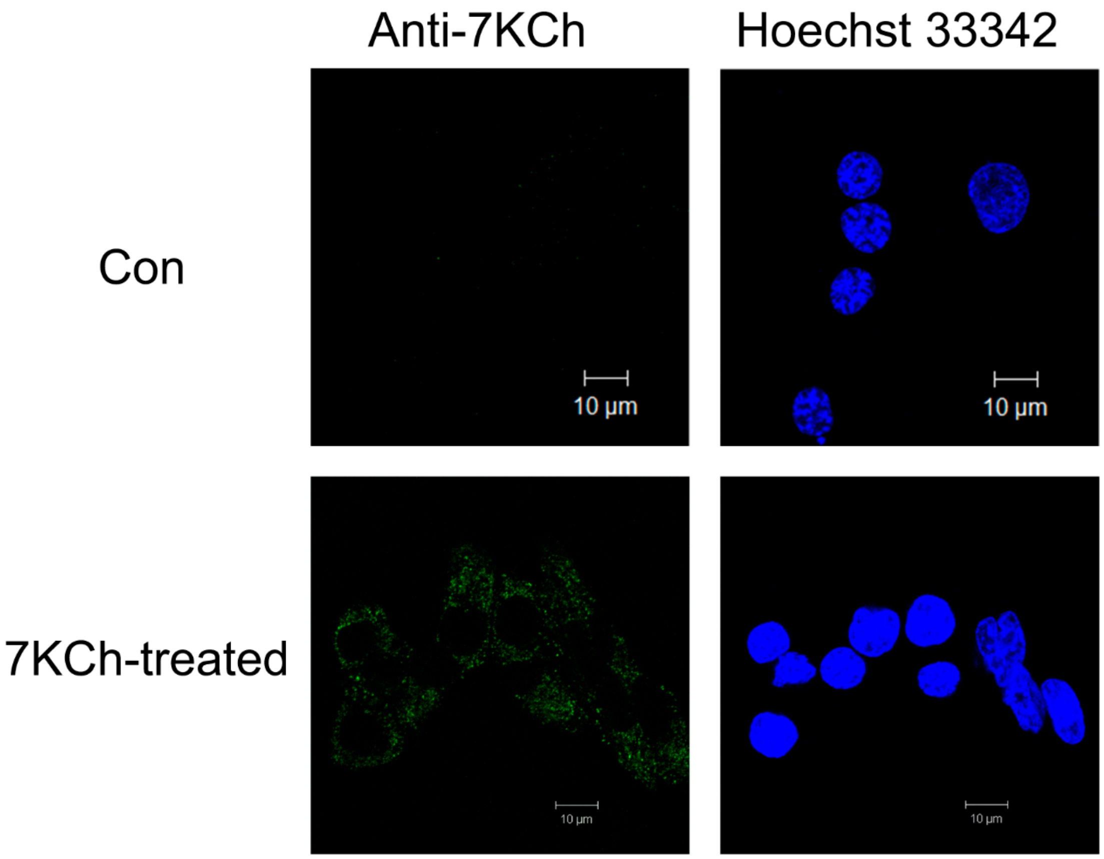

Figure S1. Accumulation of 7KCh in HL-1 cells. HL-1 cells were treated with 0 (i.e. the vehicle control; *Con*) or 20 μM 7KCh (*7KCh-treated*), and stained with the primary antibody to 7KCh and the secondary antibody conjugated to anti-mouse Dylight 488 secondary antibody. The cells were counterstained with Hoechst 33342. A representative experiment out of three is shown. Scale bar, 10 μm.

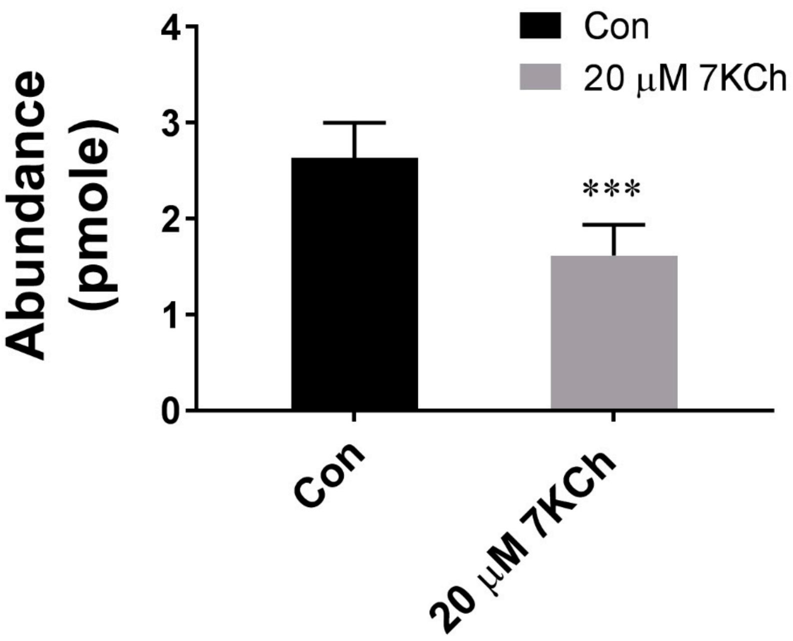

Figure S2. 7KCh treatment diminishes the CoQ10 pool. HL-1 cells were treated with 0 (i.e. the vehicle control; *Con*) or 20 μM 7KCh for 24 h, and harvested for determination of the CoQ10 content. Data are expressed in pmole per 10^5 cells, and are mean ± SD, N = 3. ***$p < 0.005$ vs. the untreated control.

Table S1. Primers used in the present study

Gene	F/R	Sequence
Cpt1a	F	5'-TGGCATCATCACTGGTGTGTT-3'
	R	5'-GTCTAGGGTCCGATTGATCTTTG-3'
Cpt1b	F	5'-GCACACCAGGCAGTAGCTTT-3'
	R	5'-CAGGAGTTGATTCCAGACAGGTA-3'
Cpt1c	F	5'-TCTTCACTGAGTTCCGATGGG-3'
	R	5'-ACGCCAGAGATGCCTTTTCC-3'
ACAC (universal primer)	F	5'-TTCCTCAACTTTGTGCCCAC -3'
	R	5'-AGGCTGATGTCCAAGTAGTAG-3'
Acaca	F	5'-ATGGGCGGAATGGTCTCTTTC-3'
	R	5'-TGGGGACCTTGTCTTCATCAT-3'
Acacb	F	5'-CGCTCACCAACAGTAAGGTGG-3'
	R	5'-GCTTGGCAGGGAGTTCCTC-3'
Acsf3	F	5'-AGGAGTGGAAGTACGCATCAT-3'
	R	5'-AACCCTGGAGTCACCTTTGTC -3'
Mlycd	F	5'-GCACGTCCGGGAAATGAAC-3'
	R	5'-GCCTCACACTCGCTGATCTT-3'
Gapdh	F	5'-AGGTCGGTGTGAACGGATTTG-3'
	R	5'-TGTAGACCATGTAGTTGAGGTCA-3'

ACAC (universal primer): the primer set for quantifying the transcript levels of variants of *Acaca* and *Acacb* genes; F/R: forward or reverse primer.
